# Supplementary material for: MetaRibo-Seq measures translation in microbiomes
Source: Nat Commun. 2020 Jun 29;11:3268. doi: 10.1038/s41467-020-17081-z (PMC7324362; doi:10.1038/s41467-020-17081-z)
Supplement: Supplementary file 10 — Supplementary Data 7 [file 41467_2020_17081_MOESM10_ESM.zip › File2/Confidence_VeryHigh_Taxonomy/5989_out.krona.html]

Javascript must be enabled to view this page.

members
magnitude
magnitudeUnassigned
count
unassigned
taxon
rank

5989\_out

5

1
2
5
superkingdom

SRS019496\_contig\_number\_contig-100\_114.88100

2
1239
phylum

class
2
186801

order
186802
2

family
186806
2

genus
1730
2

39485
2
species

SRS044535\_contig\_number\_15342SRS146764\_contig\_number\_33054

2
976
phylum

class
200643
2

1
1970189
order

1
1573805
family


SRS015663\_contig\_number\_contig-100\_651.155307
1717717
1
species

order
171549
1

1
2005525
family

genus
375288
1


SRS1041038\_contig\_number\_contig-100\_23133.59493
46503
1
species
